# Can body mass index predict clinical outcomes for patients with acute lung injury/acute respiratory distress syndrome? A meta-analysis

**DOI:** 10.1186/s13054-017-1615-3

**Published:** 2017-02-22

**Authors:** Yue-Nan Ni, Jian Luo, He Yu, Yi-Wei Wang, Yue-Hong Hu, Dan Liu, Bin-Miao Liang, Zong-An Liang

**Affiliations:** 1 Departments of Respiratory Diseases, West China School of Medicine and West China Hospital, Sichuan University, No. 37 Guoxue Alley, Chengdu, 610041 Sichuan China; 2 Departments of Critical Care Medicine, West China School of Medicine and West China Hospital, Sichuan University, 37 Gue Xue Xiang, Chengdu, 610041 Sichuan China

**Keywords:** Respiratory distress syndrome, Adult, Body mass index, Obese, Mortality, Prognosis

## Abstract

**Background:**

The effects of body mass index (BMI) on the prognosis of acute respiratory distress syndrome (ARDS) are controversial. We aimed to further determine the relationship between BMI and the acute outcomes of patients with ARDS.

**Methods:**

We searched the Pubmed, Embase, Medline, Cochrane Central Register of Controlled Trials (CENTRAL), and ISI Web of Science for trials published between 1946 and July 2016, using “BMI” or “body mass index” or “overweight” or “obese” and “ARDS” or “ALI” or “acute respiratory distress syndrome” or “acute lung injury”, without limitations on publication type or language. Heterogeneity and sensitivity analyses were conducted, and a random-effects model was applied to calculate the odds ratio (OR) or mean difference (MD). Review Manager (RevMan) was used to test the hypothesis using the Mann-Whitney *U* test. The primary outcome was unadjusted mortality, and secondary outcomes included mechanical ventilation (MV)-free days and length of stay (LOS) in the intensive care unit (ICU) and in hospital.

**Results:**

Five trials with a total of 6268 patients were pooled in our final analysis. There was statistical heterogeneity between normal-weight and overweight patients in LOS in the ICU (*I* ^2^ = 71%, *χ* ^2^ = 10.27, *P* = 0.02) and in MV-free days (*I* ^2^ = 89%, *χ* ^2^ = 18.45, *P* < 0.0001). Compared with normal weight, being underweight was associated with higher mortality (OR 1.59, 95% confidence interval (CI) 1.22, 2.08, *P* = 0.0006), while obesity and morbid obesity were more likely to result in lower mortality (OR 0.68, 95% CI 0.57, 0.80, *P* < 0.00001; OR 0.72, 95% CI 0.56, 0.93, *P* = 0.01). MV-free days were much longer in patients with morbid obesity (MD 2.64, 95% CI 0.60, 4.67, *P* = 0.01), but ICU and hospital LOS were not influenced by BMI. An important limitation of our analysis is the lack of adjustment for age, sex, illness severity, comorbid illness, and interaction of outcome parameters.

**Conclusions:**

Obesity and morbid obesity are associated with lower mortality in patients with ARDS.

**Electronic supplementary material:**

The online version of this article (doi:10.1186/s13054-017-1615-3) contains supplementary material, which is available to authorized users.

## Background

Acute respiratory distress syndrome (ARDS) is an acute, diffuse, and inflammatory lung injury that leads to increased pulmonary vascular permeability, increased lung weight, and loss of aerated tissue [1]. Clinical hallmarks of ARDS are hypoxemia and bilateral radiographic opacities, which result from the pathogenesis of diffuse alveolar damage. It was first described in 1967 as a life-threatening organ failure due to several pulmonary and extrapulmonary injuries with an incidence of 86.2 per 100,000 patient years and in-hospital mortality of up to 40% [2]. Despite intense investigations and numerous large-scale clinical trials, no specific therapies or medications have yet been developed. Although various processes of care interventions, such as lung-protective ventilation, prone positioning, and neuromuscular blockade, are proposed to be of potential benefit, mortality still remains as high as 31% [3–5]. Thus, prognostic predictors of ARDS may exert a role in helping clinicians to evaluate disease severity and make optimal treatment decisions.

Body mass index (BMI) is one of the common clinical demographic characteristics and can be calculated from the ratio of body weight to squared height (kg/m^2^). According to the definition of the National Institutes of Health (NIH), obesity can be classified into different categories on the basis of BMI: overweight (BMI ≥25 to <30 kg/m^2^), obese (BMI ≥30 to <40 kg/m^2^), or morbidly obese (BMI ≥40 kg/m^2^), compared to normal weight (BMI ≥18.5 < 25 kg/m^2^) and underweight (BMI <18.5 kg/m^2^) [6, 7]. In the USA, it was reported that at least 25% of adults in the intensive care unit (ICU) were overweight, obese, or morbidly obese, and that this proportion was increasing [8]. Obesity is associated with increased morbidity in cardiovascular disease, diabetes mellitus, and depression [9–11], which eventually leads to more than 110,000 obesity-related excess deaths annually [12] and estimated healthcare costs representing 5.7% of national health expenditure in the USA.

Pepper and colleagues report that among critically ill patients with sepsis or septic shock, patients who are overweight or obese according to BMI may have reduced risk of mortality [13]. In patients with traumatic brain injury, however, obesity tends to be associated with more complications and greater mortality [14]. Similarly, there have been controversial results in patients with ARDS in different trials. Hence, a pooled analysis of the effects of BMI on the prognosis of ARDS is warranted. Therefore, we conducted a meta-analysis of all published trials, aiming to identify the relationship between BMI and the acute outcomes of patients with ARDS.

## Methods

### Search strategies

A comprehensive computer search was conducted in Pubmed, Embase, Medline, Cochrane Central Register of Controlled Trails (CENTRAL) and ISI Web of Science for trials published between 1946 and July 2016, using the keywords “BMI” or “body mass index” or “overweight” or “obese” and “ARDS” or “ALI” or “acute respiratory distress syndrome” or “acute lung injury”, without limitations on publication type or language. We also reviewed the references listed in each identified article and manually searched the related articles to identify all eligible studies and to minimize the potential publication bias.

### Inclusion and exclusion criteria

Eligible clinical trials were identified based on the following criteria: (1) the subjects enrolled in each study included patients with ARDS; (2) patients were divided into underweight, normal weight, overweight, obese and morbidly obese, based on BMI; and (3) outcomes included, but were not limited to, mortality, length of stay (LOS) in the ICU, LOS in hospital, or mechanical ventilation (MV)-free days. We excluded studies if they were performed in animals or in patients who were under 18 years of age, or if they were published as reviews or case reports.

### Study selection

Two independent investigators performed the study selection in two phases. They first discarded duplicated and non-controlled studies by screening titles and abstracts. They then extracted eligible studies by reviewing full texts in accordance with the previously designed study inclusion criteria. Any disagreement was solved by mutual consensus in the presence of a third investigator.

### Data extraction

Independently, two data collectors extracted and recorded the desired information from each selected study in a standard form as recommended by Cochrane [15], which included information on authors, publication year, study design, country, participants and population, demographic characteristics (age, gender, etc.), comorbilities (diabetes mellitus, liver disease, etc.), assessments of disease (Acute Physiology and Chronic Health Evaluation (APACHE) III and Simplified Acute Physiologic Score (SAPS) II), outcome measures, and study results. For any missing data, corresponding authors were contacted via email for the full original data. Different opinions between the two data collectors were resolved by consensus or by consulting a third investigator.

### Statistical analysis

Statistical analysis was accomplished by an independent statistician using Cochrane systematic review software Review Manager (RevMan; Version 5.3.5; The Nordic Cochrane Centre, The Cochrane Collaboration, Copenhagen, 2014). We used the Mann-Whitney *U* test to verify the hypothesis and rendered statistical significance as a *Z* value and *P* value <0.05; the results were displayed in forest plots. Continuous variables were reported as mean and standard derivation (SD), while dichotomous variables were reported as frequency and proportion. An initial test for clinical, methodological and statistical heterogeneity was conducted, and we used the *χ* ^2^ test with *P* < 0.1 and *I* ^2^ > 50% to indicate significance. We also performed sensitivity analysis to substitute alternative decisions or ranges of values for decisions that were arbitrary or unclear. A random-effects model was applied in all outcome analyses regardless of the statistical heterogeneity. For continuous data we calculated mean difference (MD) and 95% CI, while for dichotomous data we calculated odds ratio (OR) and 95% CI. For the assessment of risk of bias and study quality, we used the Newcastle-Ottawa quality assessment tool (Additional file 1) [16].

## Results

**Fig. 1:**
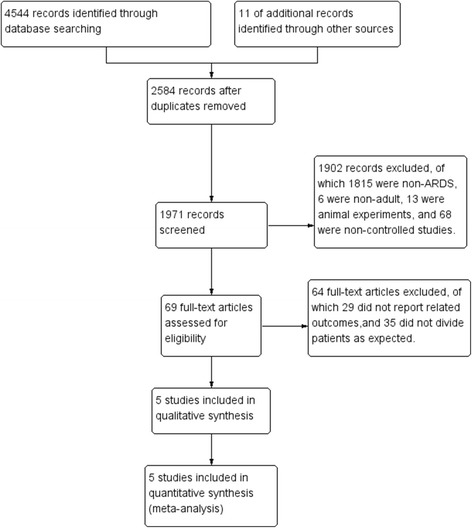
Study flow diagram

Initially 4555 records were identified, of which 4544 were extracted from electronic databases and 11 were extracted from review of reference lists (Fig. 1). By screening the titles and abstracts, we discarded 4486 studies due to duplication (*n* = 2584), non-ARDS setting (*n* = 1815), non-adult study population (*n* = 6), animal experiments (*n* = 13), or non-controlled studies (*n* = 68). We searched the full-text articles for the remaining 69 studies, and eventually 5 trials were selected for our final analysis, as 29 studies did not report related outcomes, and 35 did not categorize patients as expected.

### Study description

Each enrolled trial was approved by the corresponding institutional ethical committee (Additional file 2). All five studies examined the effects of BMI on mortality in adult ICU patients with ARDS. In one study, data on hospital and ICU mortality were reported [17]. In two studies, data on ICU mortality were provided [18, 19]. In the two remaining studies, one study presented data on 90-day mortality and the other reported data on mortality [20, 21]. Four studies reported ICU and hospital LOS [17–19, 21], while three studies provided data on MV-free days [18–20]. Details of each study included in our analysis, including different types of reporting of mortality, are summarized in Table 1.

**Table 1:** Details of each enrolled study

Author (year)	Study design	Country	Population, *n*	Outcome^a^	BMI measurement	Timing	Number with BMI/total patients (%)	Mortality data included in analysis
O’Brien 2006 [17]	Retrospective cohort, multicenter	USA	1488	①②③	Based on recorded weight and height	At ICU admission	1488/1673 (89%)	Hospital mortality
Morris 2007 [21]	Prospective cohort, multicenter	USA	825	①②③⑤	Based on recorded weight and height	At Hospital admission	825/1113 (74%)	Mortality
Stapleton 2010 [20]	Retrospective, multicenter	USA	1409	①②③④⑥	Based on recorded weight and height	At ICU admission	1409/1451 (97%)	90-day mortality
Soto 2012 [19]	Retrospective, multicenter	USA	751	①②③④⑧⑨	Based on recorded weight and height	At ICU admission	751/848 (88%)	ICU mortality
Gong 2016 [18]	Cohort, multicenter	USA	1795	①②③④	Based on recorded weight and height	At ICU admission	1795/1995 (90%)	ICU mortality

*BMI* body mass index, *ICU* intensive care unit

^a^Outcome measures include: ① mortality; ② length of stay in ICU; ③ length of stay in hospital; ④ mechanical-ventilation-free days; ⑤ duration of mechanical ventilation; ⑥ organ-failure-free days; ⑦ plasma cytokine level; ⑧ time to death; ⑨ time to acute kidney injury

All the five studies included in our analysis were multicenter studies from the USA [17–21]. BMI was recorded for 74–97% of the total study population [17–21], with BMI calculated upon ICU admission in four of these studies [17–20] and at hospital admission in the remaining study [21].

A total of 6268 patients with ARDS were pooled from all the included trials in our final meta-analysis. The majority of patients enrolled in the studies were men (51.5–63.3%), and the mean age ranged from 49.3 to 65.3 years. Details of patients’ baseline characteristics in each study included in the analysis are shown in Table 2.

**Table 2:** Baseline characteristics of patients in each trial included in the analysis

Author (year)	BMI (kg/m^2^) categories	Age, years, median (SD)	Male, *n* (%)	APACHE III, median score (SD)	SAPS II probability of survival, % (SD)	Diabetes mellitus, *n* (%)	Liver disease, *n* (%)	Smoking, *n* (%)
O’Brien 2006 [17]	Underweight	62.4 (16.2)	41 (46.6%)	NR	0.53 (0.29)	10 (11.4%)	NR	NR
	Normal	61.0 (17.8)	307 (56.4%)		0.58 (0.28)	87 (16.0%)		
	Overweight	59.4 (16.7)	223 (55.9%)		0.59 (0.29)	98 (24.6%)		
	Obese	58.0 (16.3)	152 (46.6%)		0.59 (0.28)	113 (34.7%)		
	Morbidly obese	53.6 (14.9)	44 (33.6%)		0.68 (0.29)	52 (39.7%)		
Morris 2007 [21]	Underweight	64.7 (18.4)	28 (56.0%)	82.3 (31.5)	NR	NR	NR	NR
	Normal	61.5 (18.1)	195 (64.8%)	74.9 (29.2)				
	Overweight	58.9 (17.4)	157 (66.2%)	74.9 (30.0)				
	Obese	57.0 (15.9)	116 (63.4%)	70.3 (29.8)				
	Morbidly obese	54.7 (13.9)	26 (48.1%)	75.0 (35.1)				
Stapleton 2010 [20]	Underweight	50.2 (16.8)	36 (59.0%)	97.2 (35.2)	NR	8 (13.1%)	NR	NR
	Normal	51.6 (17.9)	316 (58.3%)	89.9 (29.8)		64 (11.8)		
	Overweight	52.1 (18.3)	267 (64.0%)	87.2 (30.1)		55 (13.2%)		
	Obese	51.0 (15.5)	172 (54.6%)	83.7 (30.5)		55 (17.5%)		
	Morbidly obese	49.3 (13.1)	23 (31.1%)	81.5 (28.6)		26 (35.1%)		
Soto 2012 [19]	Underweight	65.3 (17.5)	12 (49.0%)	80.9 (20.1)	NR	2 (6.0%)	3 (10.0%)	19 (68.0%)
	Normal	58.8 (18.8)	147 (63.0%)	75.4 (22.5)		23 (10.0%)	18 (8.0%)	142 (71.0%)
	Overweight	59.0 (18.7)	164 (69.0%)	75.7 (25.7)		45 (19.0%)	10 (4.0%)	127 (66.0%)
	Obese	56.7 (16.9)	124 (66.0%)	70.4 (21.1)		50 (27.0%)	14 (7.0%)	107 (66.0%)
	Morbidly obese	50.2 (13.9)	26 (43.0%)	69.1 (21.3)		29 (45.0%)	5 (8.0%)	43 (61.0%)
Gong 2016 [18]	Underweight	61 (20.0)	35 (42.0%)	70 (22.0)	NR	14 (17.0%)	3 (4.0%)	NR
	Normal	63 (18.0)	393 (63.0%)	70 (23.0)		113 (18.0%)	40 (6.0%)	
	Overweight	62 (17.0)	421 (70.0%)	69 (24.0)		144 (24.0%)	31 (5.0%)	
	Obese	60 (17.0)	209 (57.0%)	68 (22.0)		140 (38.0%)	17 (5.0%)	
	Morbidly obese	54 (14.0)	58 (50.0%)	67 (25.0)		52 (45.0%)	8 (7.0%)	

*BMI* body mass index, *APACHE* Acute Physiology and Chronic Health Evaluation, *NR* not reported, *SAPS* Simplified Acute Physiologic Score

### Quality assessment

**Fig. 2:**
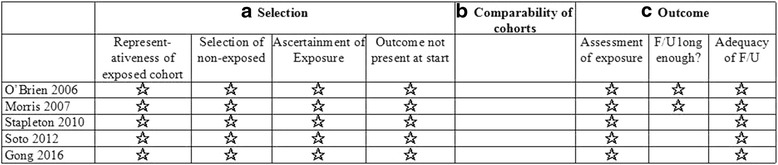
Risk of bias summary. *F/U* follow up

The Newcastle-Ottawa scale was used to assess the quality of individual studies. A maximum of 9 points was assigned to each study: 4 for selection, 2 for comparability, and 3 for outcomes. A study with a final score ≥6 was regarded as high quality. Among the five studies, two studies [17, 21] scored 7 points and three studies [18–20] scored 6 points, indicating a high risk of bias (Fig. 2).

### Heterogeneity

There was statistical heterogeneity between normal weight and overweight patients in ICU LOS (*I* ^2^ = 71%, *χ* ^2^ = 10.27, *P* = 0.02), but not between normal weight and underweight patients or between obese and morbidly obese patients. For MV-free days, there was only statistical heterogeneity between normal weight and underweight patients (*I* ^2^ = 89%, *χ* ^2^ = 18.45, *P* < 0.0001). There was no significant heterogeneity in mortality or hospital LOS.

### Mortality

**Fig. 3:**
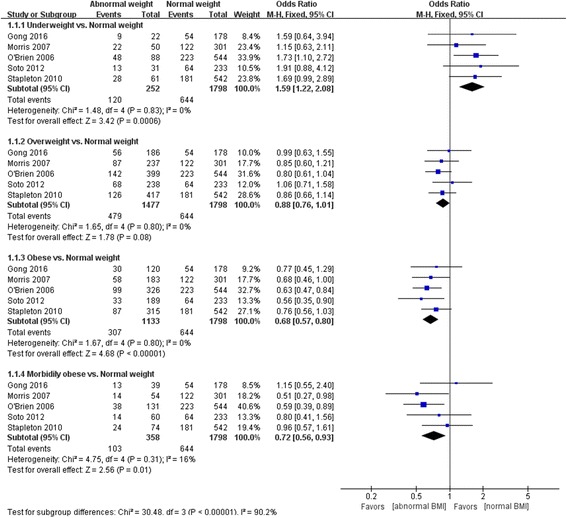
Effect of body mass index (*BMI*) on mortality. *CI* confidence interval, *SD* standard deviation

The OR of mortality in normal weight, underweight, overweight, obese, and morbidly obese patients was 1.59 (95% CI 1.22, 2.08), 0.88 (95% CI 0.76, 1.01), 0.68 (95% CI 0.57, 0.80), and 0.72 (95% CI 0.56, 0.93), respectively. There were significant differences among underweight, obese, and morbidly obese patients (*Z* = 3.42, *P* = 0.0006; *Z* = 4.68, *P* < 0.00001; *Z* = 2.56, *P* = 0.01), but not overweight patients (*Z* = 1.78, *P* = 0.08) (Fig. 3).

### MV-free days

**Fig. 4:**
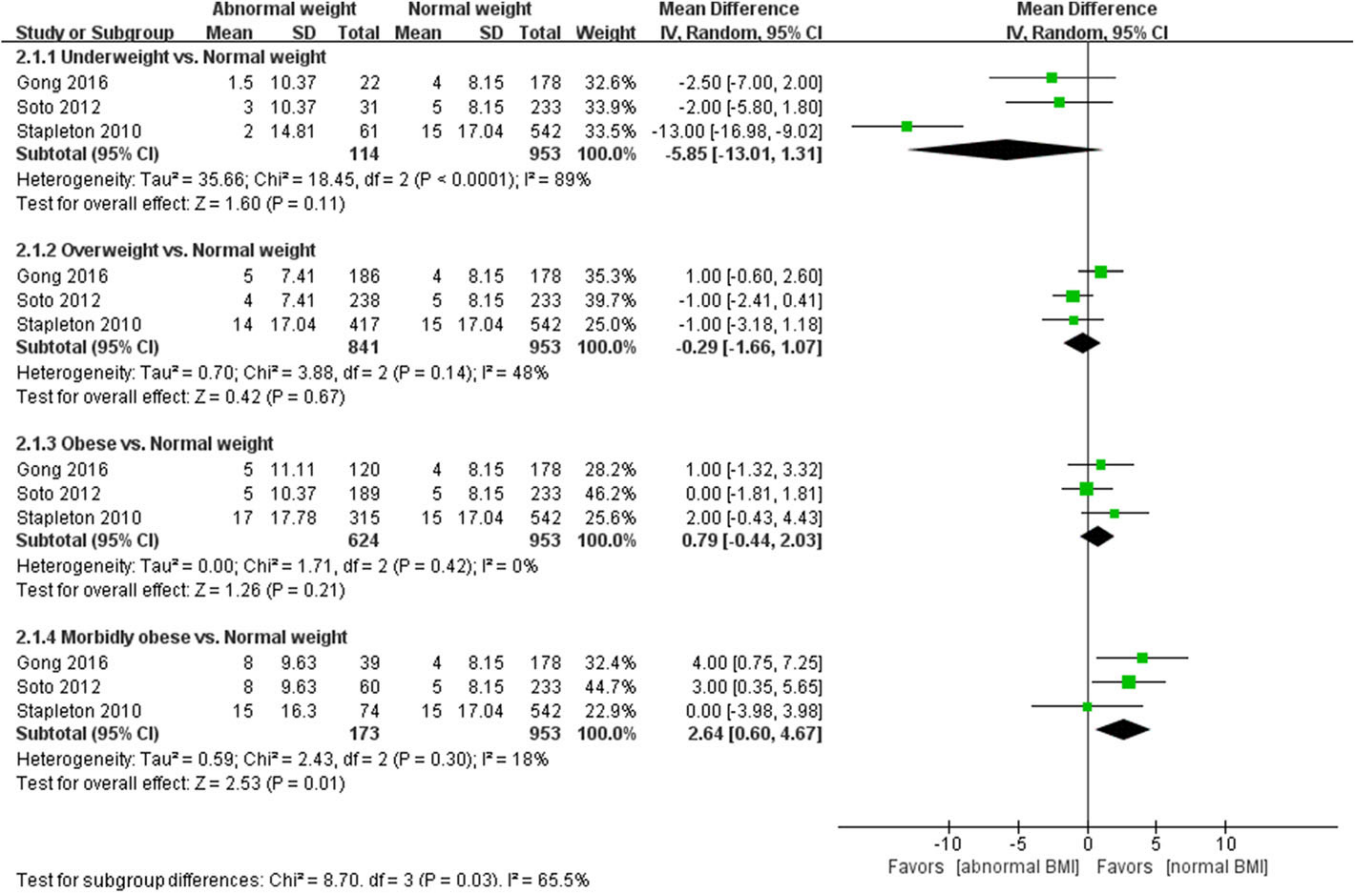
Effect of body mass index (*BMI*) on mechanical ventilation (MV)-free days. *CI* confidence interval, *SD* standard deviation

Compared with normal weight patients, the number of MV-free days was much greater in morbidly obese patients (MD 2.64, 95% CI 0.60, 4.67, *Z* = 2.53, *P* = 0.01); but there were no significant differences between normal weight and underweight (MD -5.85, 95% CI -13.01, 1.31, *Z* = 1.60, *P* = 0.11), overweight (MD -0.29, 95% CI -1.66, 1.07, *Z* = 0.42, *P* = 0.67), obese patients (MD 0.79, 95% CI -0.44, 2.03, *Z* = 1.26, *P* = 0.21) (Fig. 4).

### ICU and hospital LOS

**Fig. 5:**
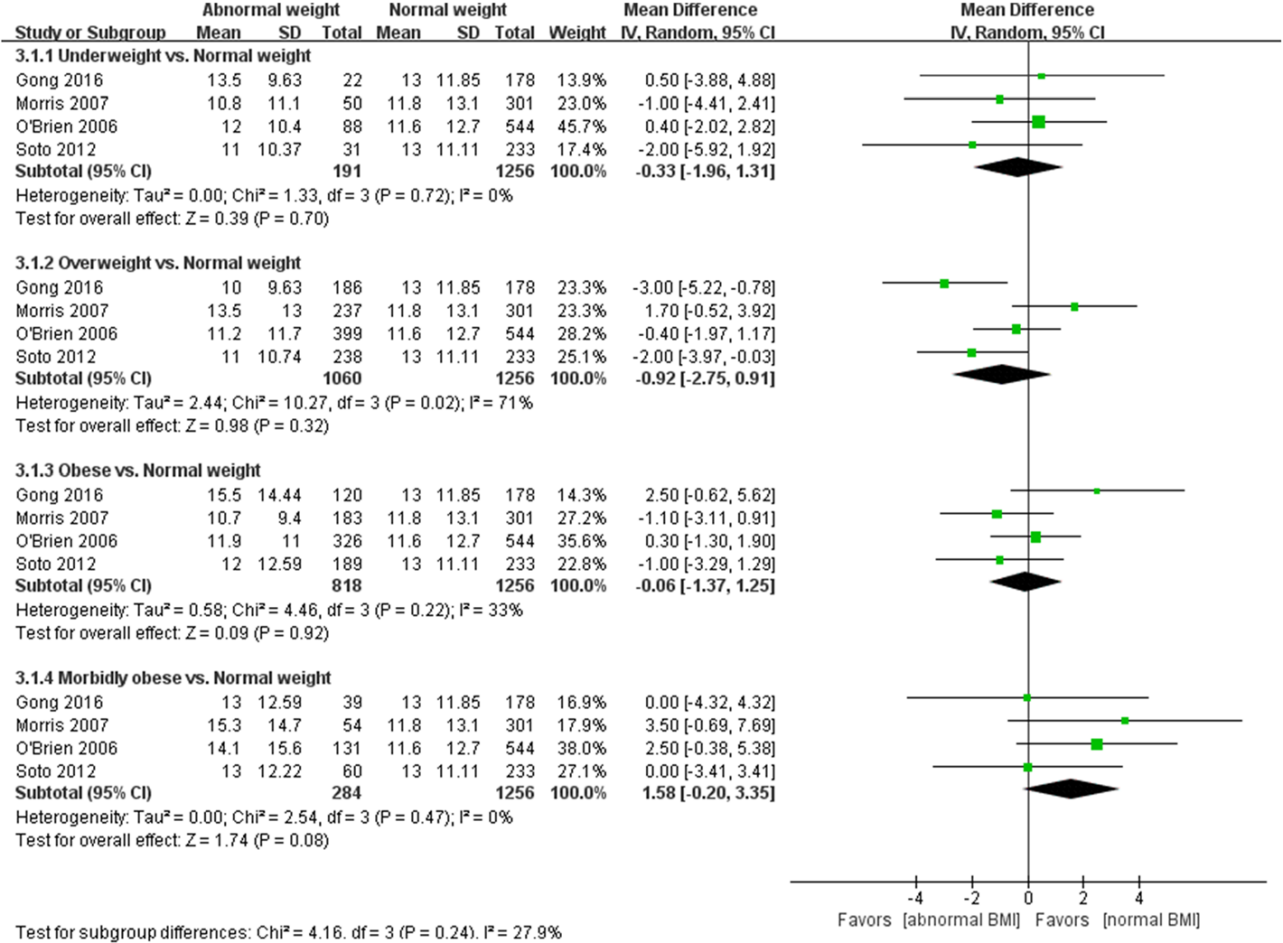
Effect of body mass index (*BMI*) on ICU length of stay (LOS). *CI* confidence interval, *SD* standard deviation

**Fig. 6:**
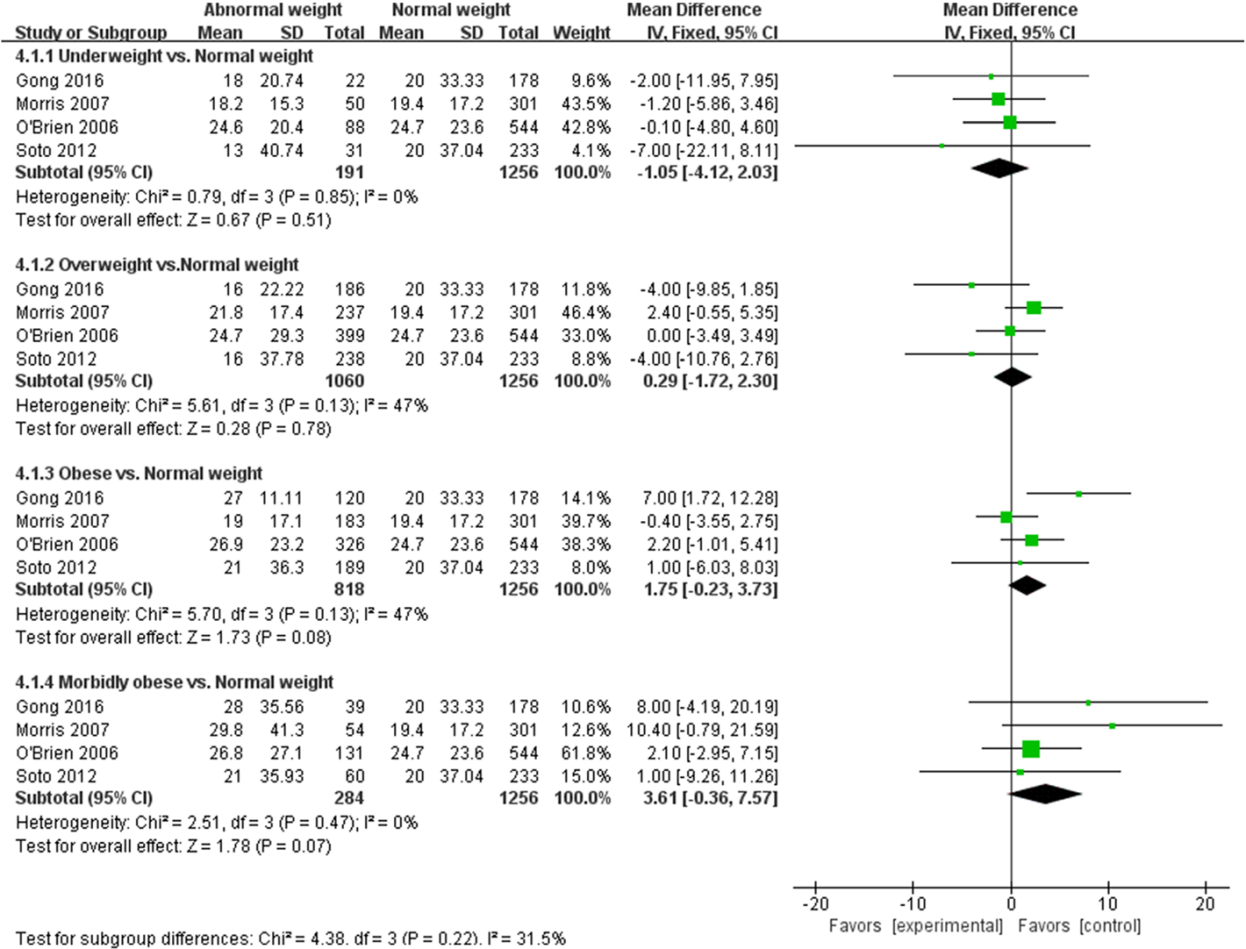
Effect of body mass index (*BMI*) on hospital length of stay (LOS). *CI* confidence interval, *SD* standard deviation

There were no significant differences between normal weight and underweight (MD -0.33, 95% CI -1.96, 1.31, *Z* = 0.39, *P* = 0.70), overweight (MD -0.92, 95% CI -2.75, 0.91, *Z* = 0.98, *P* = 0.32), obese (MD -0.06, 95% CI -1.37, 1.25, *Z* = 0.09, *P* = 0.92), or morbidly obese patients (MD 1.58, 95% CI -0.20, 3.35, *Z* = 1.74, *P* = 0.08) (Fig. 5). A similar pattern was seen in hospital LOS among different BMI groups (Fig. 6).

## Discussion

In our meta-analysis, we found that mortality was likely to lower among underweight patients with ARDS. Moreover, MV days were fewer in patients who were morbidly obese. Nevertheless, ICU and hospital LOS were not shorter in morbidly obese patients, or in obese, overweight or underweight patients.

It has been demonstrated that greater mortality in critically ill patients is associated with various clinical measurements and different severity of disease, such as older age, higher APACHE II scores, burden of comorbidity, and requirement for vasopressors [22–29]. In our meta-analysis, we noticed that morbidly obese patients had lower mortality. Previous studies report that disease was mildest among morbidly obese patients because of their younger age, lower APACHE III and SAPS II scores, higher arterial partial pressure of oxygen (PaO_2)_/fraction of inspired oxygen (FiO_2_) ratio, and lower levels of pro-inflammatory cytokines [19, 30]. Furthermore, the increased use of heparin prophylaxis among patients in the higher BMI categories mediated some protective effects for obesity and morbid obesity, such as inhibiting the coagulation phenomena to block systemic inflammatory response [21, 31]. Interestingly, hypertension, which is prevalent in obese and morbidly obese patients, was suspected to exert protective hemodynamic effects during circulatory failure and decrease the need for fluid or vasopressor support [32]. Therefore, the fact that higher BMI is a protective factor in ARDS may be reasonable. In addition, in our analysis being overweight was not shown to induce lower mortality, which we believe may be attributed to the significant statistical heterogeneity; however, further evidence and studies are still warranted.

Similarly, morbidly obese patients had shorter duration of MV in our analysis, which may be partially explained by less severe disease in patients with higher BMI. On the other hand, from the perspective of respiratory mechanics, a greater portion of the airway pressure might be allocated to distend the chest wall during inflation in patients with a higher BMI [33], thus decreasing the transpulmonary pressure. As a result, the risk of ventilator-associated lung injury will be diminished, which is further emphasized by the lower alveolar pressure due to significant smaller end-expiratory lung gas volume [34]. Moreover, it is believed that a substantial amount of lung is still hyperinflated despite a “protective” tidal volume, and a further reduction in tidal volume may be beneficial.

O’Brien reported that morbidly obese patients were often ventilated with significantly higher tidal volume based on the predicted body weight compared with patients of normal body weight, and that the need for greater tidal volume might not be physiological [35]. If the tidal volume is standardized by an identical study protocol, morbidly obese patients will receive the same tidal volume as the normal weight patients, but such a tidal volume is relatively smaller than that calculated by the individually predicted body weight. Accordingly, extra lower ventilation is applied as compared with that recommended by “lung protective ventilation”, which is termed as “ultraprotective ventilation” [36].

Nevertheless, we did not identify such a protective role of being overweight or obese in reducing the number of MV-free days, which seemed to be paradoxical and unaccountable. The effect of obesity on the duration of MV has been controversial in previous studies [37–40]. In clinical practice physicians need to be cautious in weaning overweight and obese patients off MV, especially in the absence of standardized weaning protocols, because of the perception that these patients are at a higher risk of failure compared with normal weight patients, which eventually results in delayed weaning and prolonged MV [21].

There was no significant association between BMI and ICU or hospital LOS, even though days of MV were fewer and mortality lower in morbidly obese patients. It is undeniable that medical resources and expenditure are tightly related to disease outcomes, such as bed availability in general wards and insurance status, which to some extent may offset the positive effects of higher BMI. However, the results in our study should be interpreted cautiously due to the potential significant statistical heterogeneity. For this reason, more studies focusing on this issue are necessary to draw a definite conclusion.

We think that the high heterogeneity between the overweight and normal weight group in ICU LOS may have resulted from (1) variation in BMI at different time points. Among the five studies included, Morris and colleagues estimated patients’ BMI upon hospital admission, while in the other four studies it was assessed upon ICU admission. The gap in between might have potentially affected the BMI categories, due for example, to fluid resuscitation treatment, and (2) the heterogeneous population. The proportion of overweight male patients was smaller than the proportion of normal weight patients in our included studies, except for the studies by Morris and Soto. In terms of the high heterogeneity between underweight and normal weight patients in the number of MV-free days, the different PaO_2_/PaCO_2_ in individual studies might have contributed to the heterogeneity. In the study of Stapleton, there was a higher PaO_2_/PaCO_2_ in the underweight compared with the normal weight group. By contrast, the PaO_2_/PaCO_2_ ratio was significantly lower in the underweight group in the other studies.

Our study has four limitations that need to be addressed. First, the rates of missing data in the studies included were relatively high (3–33%), which may have influenced the accuracy of our results. Second, BMI was partly calculated on the basis of physician-estimated or patient-reported weight and height, especially in the retrospective studies; this may be biased by the resuscitation fluids given prior to ICU admission, which may in turn affect patient classification. Third, there was significant statistical heterogeneity in some outcomes, complicated by a lack of risk adjustment for differences in patients, in variables such as age, sex, severity of disease (which could be seen from the APACHE III scores, SAPS scores and the value of oxygenation index), comorbid illness (such as diabetes mellitus), smoking, MV parameters, and interaction of outcome parameters. This would limit the general application of our findings.

We planned to report adjusted data when we designed our study but were unable to do so. This was because the adjusted OR and 95% CI, which are indispensable for performing meta-analysis, were only provided in one study and we did not have access to the raw data. Moreover, two of the studies included only reported ICU mortality, which may lead to underestimation of mortality because the patients may have died in the general ward after leaving the ICU, or might have been readmitted to the ICU and died during this process. Finally, none of the studies described whether treatment strategies were standardized, which may further result in bias.

## Conclusions

In patients with ARDS, obesity and morbid obesity are associated with lower mortality, which can be considered as protective factors in ARDS/ALI. There is a need for more large studies, in particular prospective studies designed with identically defined treatment protocols, and with BMI and other demographic and clinical information reported in all patients, to further determine the precise roles of BMI in patients with ARDS.

## Additional files

Additional file 1: Assessment of risk of bias and study quality. (DOC 18 kb)
Additional file 2: Names of ethical bodies of each included study. (DOC 15 kb)

## Abbreviations

ALI: Acute lung injury
APACHE: Acute Physiology and Chronic Health Evaluation
ARDS: Acute respiratory disease syndrome
BMI: Body mass index
CENTRAL: Cochrane Central Register of Controlled Trails
CI: Confidence Interval
FiO_2_: Fraction of inspired oxygen. ICU, Intensive care unit
ISI: Information Sciences Institute
LOS: Length of stay
MD: Mean difference
MV: Mechanical ventilation
NIH: National Institutes of Health
OR: Odds ratio
PaO_2_: Arterial partial pressure of oxygen
SAPS: Simplified Acute Physiologic Score
SD: Standard derivation
