# Supplementary material for: Can body mass index predict clinical outcomes for patients with acute lung injury/acute respiratory distress syndrome? A meta-analysis
Source: Crit Care. 2017 Feb 22;21:36. doi: 10.1186/s13054-017-1615-3 (PMC5320793; doi:10.1186/s13054-017-1615-3)
Supplement: Additional file 2: — Names of ethical bodies of each included study. (DOC 15 kb) [file 13054_2017_1615_MOESM2_ESM.doc]

| **Additional file 2 Names of ethical bodies of each enrolled study** | |
| --- | --- |
| Author(Year) | The specific names of ethical bodies |
| Stapleton 2010 | The University of Vermont Committee on Human Research in the Medical Sciences |
| Soto 2012 | The human subjects committees of the MGH, BIDMC and Harvard School of Public Health. |
| O’Brien 2006 | The Ohio State University Institutional Review Board |
| Morris 2007 | The University of Washington institutional review board |
| Gong 2016 | The human subjects Committees of the MGH, BIDMC and Harvard School of Public Health |
